# Properties of Foamed Mortar Prepared with Granulated Blast-Furnace Slag

**DOI:** 10.3390/ma8020462

**Published:** 2015-01-30

**Authors:** Xiao Zhao, Siong-Kang Lim, Cher-Siang Tan, Bo Li, Tung-Chai Ling, Runqiu Huang, Qingyuan Wang

**Affiliations:** 1 College of Environment and Civil Engineering, Chengdu University of Technology, Chengdu 610019, Sichuan, China; E-Mails: zhaoxiao@cdut.cn (X.Z.); hrq@cdut.edu.cn (R.H.); 2 Faculty of Engineering and Science, Universiti Tunku Abdul Rahman, Kuala Lumpur 53100, Malaysia; E-Mail: siongkang@hotmail.com; 3 Faculty of Civil Engineering, Universiti Teknologi Malaysia, Johor Bahru 81310, Malaysia; E-Mail: tcsiang@utm.my; 4 Construction and Building Materials Research Center, Nano and Advanced Materials Institute Limited, the Hong Kong University of Science and Technology, Clear Water Bay, Kowloon, Hong Kong 999077, China; E-Mail: boli@nami.org.hk; 5 Institute for Mechanics of Advanced Materials and Structures, Chengdu University, Chengdu 610106, Sichuan, China; E-Mail: wangqy@scu.edu.cn

**Keywords:** foamed mortar, water-to-cement ratio, curing regime, slag, strength, thermal properties

## Abstract

Foamed mortar with a density of 1300 kg/m^3^ was prepared. In the initial laboratory trials, water-to-cement (w/c) ratios ranging from 0.54 to 0.64 were tested to determine the optimal value for foamed mortar corresponding to the highest compressive strength without compromising its fresh state properties. With the obtained optimal w/c ratio of 0.56, two types of foamed mortar were prepared, namely cement-foamed mortar (CFM) and slag-foamed mortar (SFM, 50% cement was replaced by slag weight). Four different curing conditions were adopted for both types of foamed mortar to assess their compressive strength, ultrasonic pulse velocity (UPV) and thermal insulation performance. The test results indicated that utilizing 50% of slag as cement replacement in the production of foamed mortar improved the compressive strength, UPV and thermal insulation properties. Additionally, the initial water curing of seven days gained higher compressive strength and increased UPV values as compared to the air cured and natural weather curing samples. However, this positive effect was more pronounced in the case of compressive strength than in the UPV and thermal conductivity of foamed mortar.

## 1. Introduction

Foamed concrete is classified as light-weight concrete, in which air voids are trapped in mortar by a suitable foaming agent [1]. The foamed concrete is manufactured by blending slurry (cement paste or mortar) with pre-formed stable foam (prepared separately by aerating a foaming agent solution). The characteristics of the foamed concrete are linked to its fluidity, low self-weight, and excellent thermal and sound insulation properties. A wide range of densities (400–1600 kg/m^3^) of foamed concrete can be achieved by adjusting the dosage of pre-formed foam. Such superior characteristics have made foamed concrete a good choice for wall panel construction material since being lightweight and insulating in terms of temperature and sound are always the top priority.

Many researchers have studied the use of fly ash to produce foamed concrete due to its contribution to long-term strength [2,3,4]. The appropriate use of fly ash in foamed concrete can result in a higher ratio of strength to density [5] and can reduce the peak temperature due to its lower specific heat capacity [6]. However, relatively few detailed studies have been reported on the use of granulated blast-furnace slag as a cement replacement material in the production of foamed concrete [7]. The traditional usage of slag for cement replacement in normal concrete was found to improve the fresh and strength properties of concrete [8].

The objective of this study was to assess the feasibility of using slag as cement replacement for the production of foamed mortar. The experimental program was divided into two series. The first series was to determine an optimal w/c ratio corresponding to the highest strength among the trial mixes without compromising the fresh state properties of foamed mortar. In the second series, 50% of slag was used as a direct replacement for cement, and thereby to assess its compressive strength, ultrasonic pulse velocity and thermal conductivity properties. Only the obtained optimal w/c was applied in Series 2. The effects of different curing conditions on the properties of slag-foamed mortar were investigated and compared with the control cement-foamed mortar.

## 2. Experimental Work

### 2.1. Materials

Cement complying with Type I Portland cement per ASTM C150 [9] was used as the primary cementing material in this study. Ground granulated blast-furnace slag (GGBFS) obtained from YTL cement (Kuala Lumpur, Malaysia) and sourced from molten iron blast furnace slag grounded to cement fineness at 3220 cm^2^/g in Blaine was used as the supplementary cementing material. The chemical compositions and physical properties of cement and slag are presented in Table 1. Normal tap water and dried river sand having a particle size less than 600 μm were chosen for preparation of the foamed mortar. A locally available synthetic detergent with a specific gravity of 1.03 was used as the foaming agent throughout the study.

**Table 1:** Chemical compositions, physical, and mechanical properties of cement and slag.

Chemical Constituents(%)	Cement	Slag
SiO_2_	21.1	32.5
Al_2_O_3_	5.2	13.8
Fe_2_O_3_	3.1	0.2
CaO	64.4	42.9
MgO	1.1	5.8
SO_3_	2.5	-
Na_2_O	0.2	-
K_2_O	0.6	-
P_2_O_2_	<0.9	-
Carbon content	-	-
Physical Properties	-	-
Specific gravity	3.2	2.9
Fineness (% passing 45 μm)	93.0	100

### 2.2. Mix Compositions

In this study, the experimental work was divided into two series. A summary of the experimental work details is given in Table 2. For both series, the cement-to-sand (c/s) ratio was kept constant at 1 and the foaming agent was diluted with water in the ratio of 1:30 (by volume).

In Series 1, laboratory trials were carried out to determine an optimum water-to-cement (w/c) ratio within the range of 0.54 to 0.64. The fresh state properties and compressive strengths of water cured samples were tested to evaluate the performance of the foamed mortar. After initial laboratory trials, the obtained optimal w/c was applied in Series 2. Two types of foamed mortar were prepared in this series, namely cement-foamed mortar (CFM) and 50% slag-foamed mortar (SFM). In this series, the compressive strengths, ultrasonic pulse velocities and thermal conductivity properties were determined for foamed mortar samples cured under different curing regimes.

### 2.3. Specimens Preparation

A pre-foaming (dry) method was adopted to prepare foamed mortar. Mortar slurry was first prepared by mixing together the constituent materials of cement/slag, sand, and water. Subsequently, dry foam with a density of 40 kg/m^3^ generated (by a foam generator) was added immediately to the mortar slurry and mixed until the dry foam had uniformly distributed in to the mortar slurry. The target density of the produced foamed mortar was 1300 ± 50 kg/m^3^.

**Table 2:** Summary of experimental work details in series 1 and 2.

**Series 1: Initial laboratory trials**						
**Mix details**	**c/s (in kg/m^3^)**	**Cement: slag (in kg/m^3^)**	* Foam volume (L/m^3^)	**w/c**	**Curing condition**	**Examine properties**
ILT-0.54	1 (500:500)	1:0 (500:0)	550	0.54	Water	1. Fresh state properties (table flow spread and self-compacting slump spread) 2. Compressive strength
ILT-0.56			523	0.56		
ILT-0.58			502	0.58		
ILT-0.60			480	0.60		
ILT-0.62			465	0.62		
ILT-0.64			448	0.64		
**Series 2: Investigation on the effect of slag and curing conditions**						
**Mix details**	**c/s (in kg/m^3^)**	**Cement: slag (in kg/m^3^)**	**Foam volume (L/m^3^)**	**w/c**	**Curing condition**	**Examine properties**
CFM-A	1 (500:500)	1:0 (500:0)	518	0.56	28 Air	1. Compressive strength 2. Ultrasonic pulse velocity 3. Thermal conductivity
CFM-NW					7 Water + 21 Air	
CFM-W+A					28 NW	
CFM-W+NW					7 Water + 21 NW	
SFM-A		0.5:0.5 (250:250)	512		28 Air	
SFM-NW					7 Water + 21 Air	
SFM-W+A					28 NW	
SFM-W+NW					7 Water + 21 NW	

c/s = cementitious-to-sand ratio; w/c = water-to-cement ratio; NW = natural weather, ILT = Initial Laboratory Trails; CFM = Cement-foamed mortar; SFM = Slag-foamed mortar; * Foam volume (in liter per m^3^) decreased when the w/c ratio increased.

The fresh foamed mortar was poured into cube and panel steel moulds with dimensions of 100 × 100 × 100 mm and 300 × 300 × 100 mm, respectively. After 24 h of casting, the specimens were demolded and subjected to different curing regimes for 28 days until the day of testing. The curing regimes adopted are listed as below:

(i) Twenty eight days of air curing in the laboratory with the constant range 30 °C ± 2 °C and an average relative humidity of 65%.
(ii) Seven days of initial water curing at a constant temperature of 26 °C ± 2 °C plus 21 days of air curing.
(iii) Twenty eight days of natural weathering outside the laboratory with the temperature ranging from 26 °C to 36 °C with relative humidity ranging from 65% to 90%.
(iv) Seven days of initial water curing at a constant temperature of 26 °C ± 2 °C plus 21 days of natural weathering.

### 2.4. Testing Methods

#### 2.4.1. Fresh State Properties

A flow table test was used to determine the consistency of the fresh mixed mortar (slurry) as described in ASTM C 1437 [10]. The fresh foamed mortar produced was first poured into an inverted slump flow cone without any compaction and vibration in accordance with ASTM C 1611 [11]. The spread (inverted slump) diameter values were measured to evaluate the flowability and consistency of the fresh foamed mortar mix.

#### 2.4.2. Compressive Strength

Compressive strength was determined by using a universal compression test machine with a constant loading rate of 0.1 kN/s in accordance with BS 4551 [12]. Three cube specimens with a size of 100 × 100 × 100 mm were tested for each mix property and curing regime.

#### 2.4.3. Ultrasonic Pulse Velocity

An ultrasonic pulse velocity (UPV) test was conducted to assess the quality of the foamed mortar by using a PUNDIT meter in accordance with BS 1881: Part 203 [13]. A total of eight points of pulse transmitted from all faces of a panel specimen were collected.

#### 2.4.4. Thermal Conductivity

**Figure 1:**
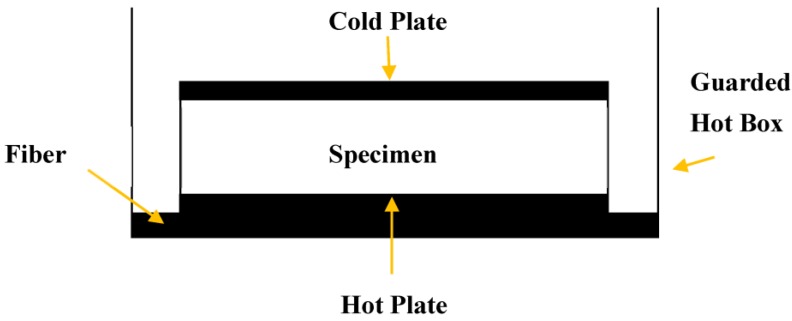
The position of the specimens inside the guarded hot box.

**Figure 2:**
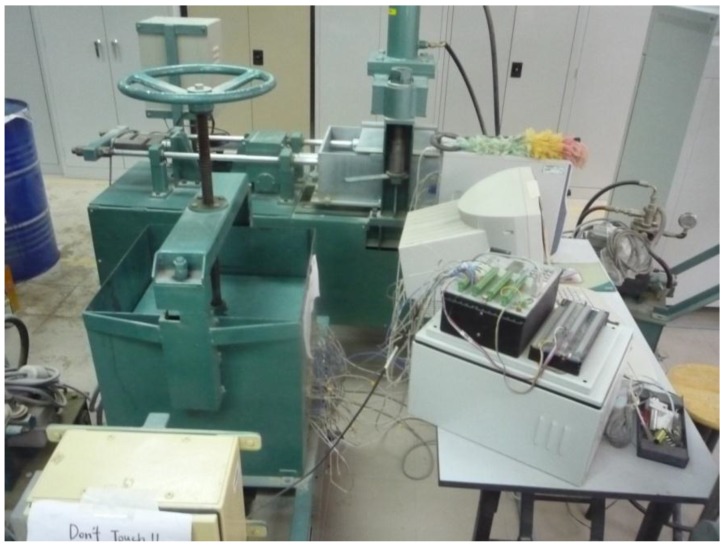
The typical setup for a thermal conductivity test.

A thermal conductivity test was conducted according to BS EN 12664 [14] in which a piece of cold plate (on top) and a piece of hot plate (on bottom) were attached to the container as shown in Figure 1. Power was supplied to the hot plate, providing a constant temperature of 40 °C while a chiller system was applied to the cold plate to maintain the temperature at 25 °C. A control unit was installed to monitor and control the temperature change. A data logger Campbell Scientific CR3000 was attached to the sealed container for data recording as shown in Figure 2. Prior to testing, specimens were dried to constant mass in a ventilated oven at 105 °C.

Thermal conductivity, *k*, is defined as the quantity of heat (*Q*) transmitted through a unit thickness (*L*) in a direction normal to a surface unit area (*A*) due to a unit temperature gradient (Δ*T*) under steady state conditions. The heat transfer is dependent only on the temperature gradient. Thermal conductivity is formulated by the equation as follows:

(1) $$k=\frac{Q\;\times \;L}{A\;\times \text{ Δ}T}$$

where *k* is the thermal conductivity (W/mK); *Q* is the heat transmitted (W); *L* is the thickness of the conducting surface separating the two temperatures (m); *A* is the total cross sectional area of conducting surface (m^2^) and Δ*T* is the temperature difference (K).

## 3. Results and Discussion

### 3.1. Initial Trial (Series 1)

The target mixture in the initial laboratory trials (Series 1) was to produce the highest compressive strength, yet being stable and flowable without compromising the fresh state properties of the foamed mortar. As a result, the effect of water-to-cement (w/c) ratios on spread (flow table) and slump (self-compacting flow) values, as well as the seven-day compressive strength were examined and compared.

**Figure 3:**
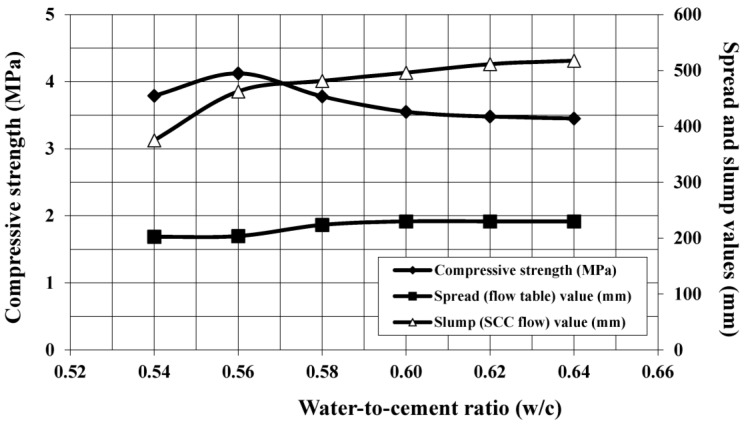
Effect of water-to-cement (w/c) ratios on spread and invert slump values, and seven-day compressive strength of series 1 foamed mortar.

From Figure 3, it is evident that all the mortar mixes (slurry) showed consistent behavior with flow table spread values within the range of 200 mm to 240 mm. It was clearly indicated that the fluidity and consistency of the mortar mix was dependent on the amount of water in the mix. The spread increased gradually when the w/c ratio increased from 0.54 to 0.64. The highest spread value of 240 mm was recorded for the mortar mix with a 0.64 w/c ratio, which might be too slurry and thin to hold the bubbles leading to separation of bubble foam in the mix and, eventually, segregation.

On the other hand, the mortar mix with a 0.54 w/c ratio obtained an average spread value of 203 mm, indicating relatively lower workability and a stiffer characteristic as compared to the mixes with higher w/c ratios. This may cause the bubbles in the foam to break due to merging and overlapping, leading to loss of foam in the mix, thus increasing the foam volume to obtain the desired density.

Comparing the slump (self-compacting flow) values of each specimen prepared with different w/c ratios, the slump values increased as the w/c ratio increased. For example, the slump value for 0.54 w/c foamed mortar was 375 mm, while for 0.64 w/c foamed mortar, it was 518 mm. This indicates that the amount of water used affected the slurryness of the foamed mortar and the foam volume that was applied into the mix to achieve the target density. Based on the results in Figure 3, the inverted slump value within 450 ± 50 mm provided the optimum strengths.

The compressive strength of foamed mortar is usually associated with the flowability of fresh mix. The seven-day compressive strength results showed that the highest compressive strength (4.1 MPa) was obtained at the w/c ratio of 0.56. For 0.56 w/c foamed mortar, it was determined that the compressive strength was about 8.1% and 16.3% higher than that of 0.54 and 0.64 w/c ratio foamed mortar, respectively. In addition, as the fresh mix at this w/c ratio was relatively stable and consistent, the w/c ratio of 0.56 was chosen for the Series 2 investigations.

### 3.2. Foamed Mortar with 0.56 w/c (Series 2)

From the initial laboratory trials (Series 1), the optimal w/c ratio of 0.56 was used in Series 2. Two types of foamed mortar, cement-foamed mortar (CFM) and 50% slag-foamed mortar (SFM), were prepared to further investigate the properties of compressive strength, ultrasonic pulse velocity and thermal conductivity under different curing conditions. The average slump flow values obtained for CFM and SFM were 490 mm and 460 mm, respectively. The relatively lower slump flow value of SFM was probably due to the fineness of GGBFS used.

#### 3.2.1. Compressive Strength

**Figure 4:**
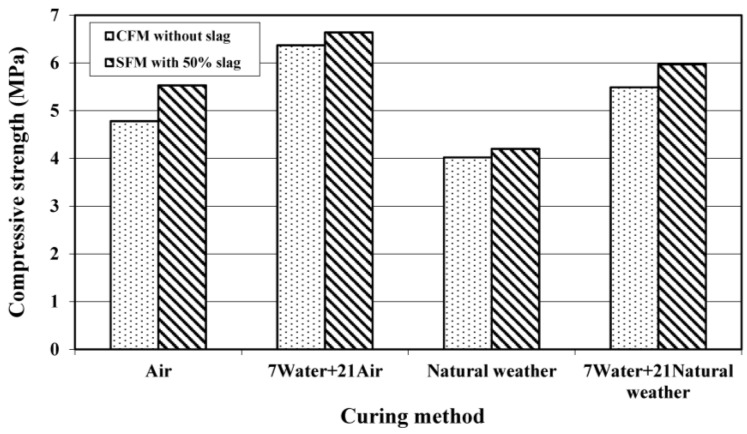
Effect of curing conditions on 28-day compressive strength of foamed mortar.

The effect of curing conditions on the 28-day compressive strength for both CFM and SFM is illustrated in Figure 4. It is worth noting that the SFM gained a higher strength than that of CFM, regardless of the curing regime. This may be due to the hydration of slag, which formed blockages in the pore capillaries, causing more air to be trapped and a resulting higher compressive strength. Similar results have been reported by Durack and Weiqing [15] on the effect of pozzolanic material on strength. They stated that the development of strength was due to the strong interring particle bond between fly ash particles and gel matrix.

In terms of curing conditions, the highest strength of foamed mortar was obtained under the seven days of initial water curing plus 21 days of air curing (7 W + 21 A) conditions. The compressive strength of SFM and CFM cured under such conditions (7 W + 21 A) were 6.4 MPa and 6.6 MPa, respectively. Another curing regime with initially seven days of water curing, (7 W + 21 NW) also provided a compressive strength of 6.0 MPa and 5.5 MPa for their respective SFM and CFM specimens. In fact, the seven days of initial water curing seemed to provide a good early hydration and led to better strength development of the foamed mortar produced.

The natural weathering regimen produced the lowest compressive strength for both types of foamed mortar. It caused an average reduction in compressive strength of 28.2% as compared to the (7 W + 21 NW) curing conditions. This is because the strength development with exposure to natural weathering was crucially dependent on the humidity and temperature changes [16,17]. However, all the foamed mortars met the minimum average compressive strength of non-structural bearing load application with an average compressive strength value of not less than 3.5 MPa in accordance with ASTM C 129 [18].

**Figure 5:**
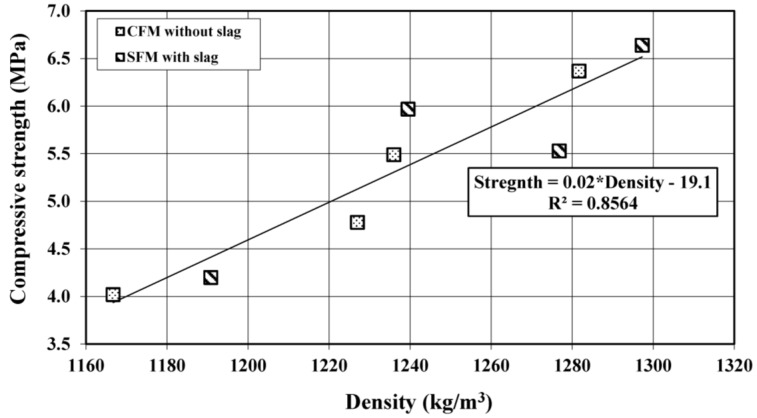
Strength change as a function of density yielded under different curing conditions.

Figure 5 shows the compressive strength change with density yielded under different curing conditions for both CFM and SFM foamed mortar. As can be seen, the compressive strength was well correlated with the density, regardless of the type of foamed mortar. The strength increased when the density increased. For instant, the strength increased from 4.02 to 6.64 MPa as the density increased from 1167 to 1293 kg/m^3^. By using the correlated equation shown in Figure 5: Strength = 0.02 × Density − 19.1, the 28-day compressive strength of foamed mortar can be predicted, provided that the density is within the tested range.

#### 3.2.2. Ultrasonic Pulse Velocity

Ultrasonic pulse velocity (UPV) is one of the most popular non-destructive techniques used for assessing concrete properties. Although it is not an accurate tool to measure the pore structures, it can still give a good preliminary prediction of the quality of a mortar. Since the UPV values could be affected by various parameters, the following discussions on UPV will only consider the affecting factors of curing conditions and slag used.

**Figure 6:**
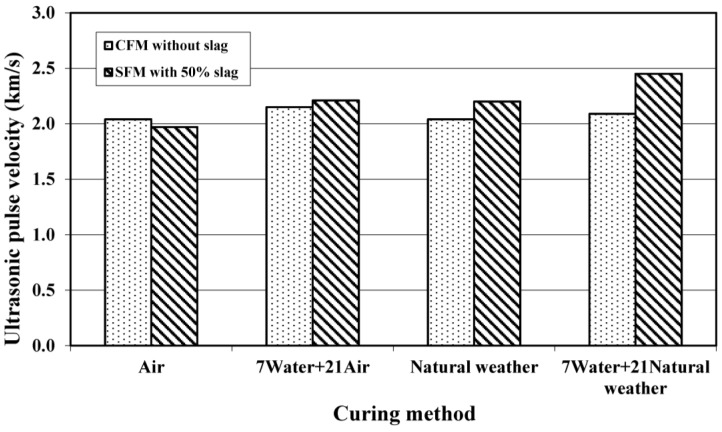
Effect of curing conditions on 28-day ultrasonic pulse velocity of foamed mortar.

Figure 6 shows the UPV results of CFM and SFM specimens under different curing conditions. For a given curing regimen, the UPV of SFM specimens was slightly higher than that of CFM specimens. This is consistent with the results of strength shown in Figure 4. This might be related to the hydration mechanism of the binary cementitious system. The hydration products formed by alkalis and silicates tended to contribute to the refinement of the pore network, leading to smaller gel pores in the cement paste. Furthermore, due to the pozzolanic reaction and filler characteristics of slag, the density of microstructures in the foamed mortar increased.

The foamed mortar exposed to seven days of initial water curing plus 21 days of natural weathering obtained the highest value of UPV. For the SFM and CFM specimens cured under these conditions (7 W + 21 NW), the average value of 2.45 km/s and 2.09 km/s were measured, respectively. The possible reason could be the same as that observed in the case of compressive strength. The initial seven days of water curing enable continuous hydration at an early stage and thus more bubbles were trapped, making the foamed mortar denser and allowing the wave to be transmitted at a faster rate through the solid mortar compared to mortar with larger pore capillaries.

#### 3.2.3. Thermal Conductivity

**Figure 7:**
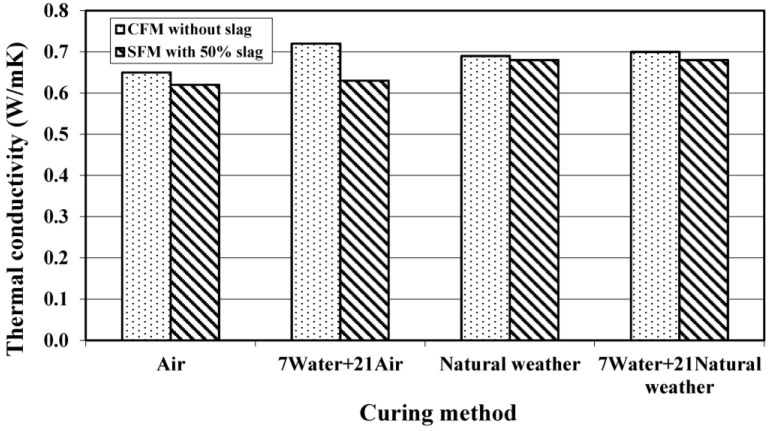
Effect of curing conditions on 28-day thermal conductivity of foamed mortar.

Figure 7 shows the thermal conductivity results of foamed mortar under different curing conditions. A small reduction in thermal conductivity is observed in foamed mortar with slag for all types of curing conditions. The percentage of reduction in the thermal conductivity of SFM specimens ranged from 1.5% to 12.5% when compared to their respective CFM specimens under the same curing conditions. The reduction in thermal conductivity (better heat insulation) could be due to the lower density and particle morphology of slag, leading to increases in heat flow paths. Another possible reason was probably related to the increase in cement fineness through the utilization of slag, since the larger interface area would act as a thermal barrier decreasing the amount of heat transfer.

The air cured slag-foamed mortar exhibited the lowest thermal conductivity. It may be due to the internal heat conductivity affected by the moisture content of the respective specimens. Nevertheless, the overall effect of curing conditions on thermal conductivity is more or less similar to that of UPV.

### 3.3. Further Discussion on the Effects of Curing Conditions

Air curing and natural weathering with insufficient moisture and fluctuation in temperature might cause incomplete hydration in foamed mortar. Thus, the foamed mortar experienced a sort of irreparable loss, leading to the degradation in strength development. Also, the development of the strength of foamed mortar was not only a function of humidity/moisture but also temperature change. When foamed mortar was subjected to a high temperature, it accelerated the early hydration process and resulted in a rapid development of strength. In contrast, the speed of the hydration process became slow during cold weather. This alternating temperature between hot and cold is found to be very common in Malaysia (tropical weather). Thus, the hydration process could be decelerated due to the decrease in temperature on rainy days. During hot weather, when the humidity/moisture concentration in the surrounding air is relatively low, the water might vaporize from the foamed mortar and lead to insufficient moisture for the hydration process.

Water curing was found to be one of the most efficient and suitable methods of curing as it satisfied most of the requirements of good curing. In the current study, it is noted that the early seven days of water curing promoted better initial hydration and compressive strength of foamed mortar. The water curing regime provided sufficient free water needed for strength development, instead of only relying on the moisture content in the mortar itself for the hydration process.

## 4. Conclusions

Based on the results obtained in this study, the following salient conclusions can be drawn:

1. The flowability of foamed mortar increased with increasing w/c ratio. However, a w/c ratio of 0.56 provided the highest compressive strength within the tested w/c ratios, ranging from 0.54 to 0.64.
2. The use of 50% slag as cement replacement in foamed mortar improved the compressive strength and ultrasonic pulse velocity as well as thermal insulation properties. The relationship between the 28-day compressive strength and density yielded under different curing conditions of foamed mortars are proposed that can be used to predict strength within the tested density range.

A comparison of the curing conditions showed that the specimens cured with seven days of initial water curing showed higher compressive strength, UPV and thermal conductivity values than those specimens cured under air and natural weather conditions. Nevertheless, the positive effect of initial water curing was more pronounced for compressive strength than that of UPV and thermal insulation of the tested foamed mortar.
