# Addendum: Zhao, X.; *et al.* Properties of Foamed Mortar Prepared with Granulated Blast-furnace Slag. *Materials* 2015, *8(2)*, 462-473

**DOI:** 10.3390/ma8073958

**Published:** 2015-07-01

**Authors:** Xiao Zhao, Siong-Kang Lim, Cher-Siang Tan, Bo Li, Tung-Chai Ling, Runqiu Huang, Qingyuan Wang

**Affiliations:** 1 State Key Laboratory of Geohazard Prevention & Geoenvironment Protection, Chengdu University of Technology, Chengdu 610019, Sichuan, China; E-Mails: zhaoxiao@cdut.cn (X.Z.); hrq@cdut.edu.cn (R.H.); 2 Faculty of Engineering and Science, Universiti Tunku Abdul Rahman, Kuala Lumpur 53100, Malaysia; E-Mail: siongkang@hotmail.com; 3 Faculty of Civil Engineering, Universiti Teknologi Malaysia, Johor Bahru 81310, Malaysia; E-Mail: tcsiang@utm.my; 4 Construction and Building Materials Research Center, Nano and Advanced Materials Institute Limited, the Hong Kong University of Science and Technology, Clear Water Bay, Kowloon, Hong Kong 999077, China; E-Mail: boli@nami.org.hk; 5 Institute for Mechanics of Advanced Materials and Structures, Chengdu University, Chengdu 610106, Sichuan, China; E-Mail: wangqy@scu.edu.cn

The authors world like to amend the following affiliation for Xiao Zhao and Runqiu Huang of paper [1]:

^1^ State Key Laboratory of Geohazard Prevention & Geoenvironment Protection, Chengdu University of Technology, Chengdu 610019, Sichuan, China

The authors apologize for any inconvenience caused to the readers.
